# Epidemic keratoconjunctivitis outbreak in a closed psychiatry ward

**DOI:** 10.1186/1753-6561-5-S6-P97

**Published:** 2011-06-29

**Authors:** P Rodriguez-Perez, NV Vendula, C-C Mireia, ZA Ana

**Affiliations:** 1 Medicina Preventiva, Hospital General Universitario Gregorio Marañon, Madrid, Spain

## Introduction

Epidemic keratoconjunctivitis (EKC) caused by adenovirus is a highly contagious infection of the eye frequently associated with healthcare services.

## Objective

Description and analysis of an outbreak of epidemic keratoconjunctivitis (EKC) in a psychiatry ward of Gregorio Marañón General University Hospital, Madrid, Spain, which occurred during the months of May and June 2010.

## Patients and methods

The outbreak occurred on a closed unit of the Psychiatry Department with 26 beds in 22 rooms. At the moment of the alert, 22 beds were occupied. The management of the outbreak consisted of reinforcement of hygiene measures and workshops on hand hygiene for the ward staff. The emergence of new cases in patients as well as in staff members lead to the restriction of new admissions and eventually to the closure of the unit.

## Results

Altogether, 13 cases of EKC were identified among patients (12 probable cases and 1 confirmed case). There were 2 probable cases identified among staff. The overall attack rate was 22.4% (13/58) among the patients and 11.7 % (2/17) among staff.

## Conclusions

As far as we know, this is the first description of an EKC outbreak in a psychiatry ward where nosocomial infections are rather rare. The psychiatric pathologies of the patients of this specific unit caused many difficulties in the reinforcement of strict preventive measures and in the therapeutic management of these patients.

## Disclosure of interest

None declared.

